# The Impact of Naturalistic Age Stereotype Activation

**DOI:** 10.3389/fpsyg.2021.685448

**Published:** 2021-07-09

**Authors:** Carla M. Strickland-Hughes, Robin L. West

**Affiliations:** ^1^Department of Psychology, University of the Pacific, Stockton, CA, United States; ^2^Department of Psychology, University of Florida, Gainesville, FL, United States

**Keywords:** age-based stereotype threat, story recall, memory, perceived threat, subjective age, memory beliefs, metamemory, stereotype priming

## Abstract

Almost self-fulfilling, commonly held negative stereotypes about old age and memory can impair older adults’ episodic memory performance, due to age-based stereotype threat or self-stereotyping effects. Research studies demonstrating detrimental impacts of age stereotypes on memory performance are generally conducted in research laboratories or medical settings, which often underestimate memory abilities of older adults. To better understand the “real world” impact of negative age and memory stereotypes on episodic memory, the present research tested story recall performance of late middle-aged and older adults (*N* = 51) following a naturalistic age stereotype manipulation, wherein every day, newspaper-style materials (comics and puzzles) were either embedded with negative age and memory stereotype stimuli (stereotype group) or neutral stimuli (control group). Furthermore, all participants were tested in favorable, familiar environments. Potential moderators of the stereotype effects, e.g., metamemory beliefs, were assessed at baseline. Current memory evaluation and subjective age, as well as perceived stereotype threat and task-related anxiety, were assessed following the stereotype manipulation as potential mechanisms of the expected stereotype effects. Results suggested a contrast effect, as the stereotype group demonstrated superior story recall performance compared to the control group. Marginally significant moderation effects by age and perceived stereotype threat indicated that stereotype rejection was present for late middle-aged adults but not older adults, indicative of stereotype lift, and for individuals who reported low and average, but not high, levels of perceived stereotype threat. Additionally, a trend suggested more positive memory evaluation for those in the stereotype group who reported awareness of the stereotype stimuli than those who did not notice the stimuli. These results are consistent with other research demonstrating benefits to memory performance in adulthood based on motivational and contextual factors, such as using relevant memory materials and testing in favorable conditions. Moreover, the results of this study contribute to our understanding of individuals’ responses to different types of stereotype stimuli, and the differential impact of stereotype manipulations that are subtle versus blatant. Individuals were motivated to counteract negative stereotype effects when conditions were supportive, stereotype presentations were naturalistic, and personal beliefs were positive.

## Introduction

Pervasive negative stereotypes about memory in aging and commonly held expectations of universal, inevitable, and irreversible senility in late adulthood (Hummert, 2011) are partly based on social truths. Age-related deficits in episodic memory are well-documented in both longitudinal and cross-sectional studies (Park and Festini, 2016; Cabeza et al., 2018). However, in adulthood, not all aspects of memory are characterized by steep declines, individuals vary greatly in their memory performance and change, and episodic memory performance can be improved via intervention and engaged lifestyles (Hertzog et al., 2008; Gross et al., 2012; Strickland-Hughes and West, 2016; Nyberg and Pudas, 2019). Furthermore, age-related deficits in episodic memory performance rarely generalize to impairments in everyday cognitive functioning (Salthouse, 2012; Barber, 2020), and laboratory and clinical settings consistently underestimate the cognitive competence of older adults (Barber and Lui, 2020). Thus, current thinking emphasizes the importance of social, motivational, and other contextual factors–not just ability–in explaining memory performance in aging (Hess and Emery, 2012; Hess, 2014; Wu and Strickland-Hughes, 2019).

For example, older adults may not be motivated to selectively engage their cognitive resources on abstract, laboratory memory tasks (Hess, 2014). Some supporting research shows older adults might better remember characteristics about people than lists of words (Sindi et al., 2013), information of high, versus low, social importance (Hargis and Castel, 2017) or positive, rather than negative, information (Mather and Carstensen, 2005). Older adults may also underperform on memory tests compared to their true competence level when tested in unfamiliar research laboratory and medical clinic settings, compared to familiar settings, such as community centers (Hehman and Bugental, 2013; Sindi et al., 2013; Eich et al., 2014; Schlemmer and Desrichard, 2018). Ironically, pervasive negative stereotypes about memory and aging themselves might be one factor that disrupts older adults’ memory performance, functioning like a science fiction “causal loop temporal paradox”: Negative age and memory stereotypes, self-held or assumed to be held by others, might worsen memory performance, reinforcing the validity of the stereotypes.

Several theories can explain the impact of age stereotypes on older adults’ memory performance. Stereotype threat theory (Steele, 1997) proposes that concern about performance judgments based on membership in a social group, and related fears of confirming the stereotypes, disrupt performance. This occurs possibly through “hot” cognitive mechanisms, such as increased anxiety, distracting thoughts, or demands on working memory (Wheeler and Petty, 2001; Pennington et al., 2016). Stereotype threat effects may be more pronounced when individuals’ highly value the stereotyped domain, perceive that performance assessment will be related to the stereotype, and strongly identify with the stereotyped group. Each of these effects has been demonstrated in memory research (Chasteen et al., 2011). Alternatively, a stereotype threat situation might elicit *stereotype reactance*, where individuals could experience increased motivational arousal and better (stereotype inconsistent) performance in response to presumed limitations on their freewill associated with being categorized into the stereotyped group (Miron and Brehm, 2006). For example, women may demonstrate “better” negotiation behaviors when threatened explicitly with gender stereotypes (Kray et al., 2004).

Individuals may also experience improved performance following the stereotype threat manipulation if they do not identify as part of the stereotyped group, due to downward social comparison. *Stereotype lift* refers to performance improvements resulting from negative stereotype activations related to a denigrated outgroup (Walton and Cohen, 2003). Meta-analytic work using the control groups from stereotype threat research indicates that non-stereotyped groups (e.g., Caucasian males) perform better (e.g., on intelligence tests) when the negative outgroup stereotype is emphasized rather than nullified (*d* = 0.34; Walton and Cohen, 2003). Importantly, several proposed mechanisms for stereotype lift are common to those for stereotype threat, such as anxiety (lower for lift), self-efficacy (higher for lift), and concerns about perceived judgment (fewer for lift).

Although stereotype threat can impact performance for many different social groups, age-based stereotype threat effects are specific to disruptions of older adults’ performance due to “old age” stereotypes (Barber, 2020). Meta-analyses confirm that age-based stereotype threat effects can impair older adults’ performance on cognitive tasks in general (*d* = 0.36; Lamont et al., 2015) and episodic memory specifically (*d* = 0.25; Armstrong et al., 2017). Notably, stereotype threat requires identification with the stereotyped group. Yet, age stereotypes are unique from stereotypes about other social groups (e.g., race, gender) given the malleability of their self-relevance and the dynamic, multidimensional nature of defining “old.” For example, middle-aged and older adults generally feel significantly younger than their chronological age (Rubin and Berntsen, 2006) and may further psychologically distance themselves from “old age” when presented with negative aging stereotypes (Weiss and Freund, 2012; Weiss and Lang, 2012). On the other hand, individuals report feeling older immediately after memory testing (Hughes et al., 2013). Further, one’s age salience and age identification may change responsively to social and contextual factors, and the transition from “middle-aged” to “old age” is defined culturally and socially (e.g., retirement, grandparenthood), not just chronologically (Montepare, 2009; Brothers et al., 2017). Thus, the delineation of “ingroup” versus “outgroup” status for “old age” stereotypes–and potential for stereotype threat versus stereotype lift effects–may be “murky,” especially for late middle-aged adults (e.g., in their 50’s or early 60’s) or young-old adults (e.g., those in their late 60’s or early 70’s). Indeed, stereotype threat effects are generally more pronounced for young-old than old-old adults (Hess et al., 2004; Eich et al., 2014). Additionally, although middle-aged adults are not commonly included in age-based stereotype threat research, an exemplar study (Hess and Hinson, 2006) compared the impact of positive and negative age stereotype activations on memory performance of adults aged 24–86 years old. They report a stereotype lift effect for middle-aged adults and stereotype threat effect for older adults, with better and worse memory performance, respectively, following the negative age stereotype presentation compared to a positive age stereotype presentation. The importance of this transitional period is highlighted in the present research with the inclusion of people over 50, that is, late middle-aged adults.

Whereas age-based stereotype threat effects traditionally focus on concerns about stereotyping by others, which assumes awareness of the stereotypes or “threat in the air” (Steele, 1997), self-stereotyping may also occur when individuals apply negative stereotypes about old age to themselves, possibly subconsciously; this can result in stereotype-consistent performance (O’Brien and Hummert, 2006). In fact, a cold cognitive account suggests stereotype priming may also result in stereotype-consistent performance for outgroup members, purely because the presentation of the stereotype makes its content more cognitively accessible and influences behavior automatically (Wheeler and Petty, 2001). Stereotype embodiment theory (Levy, 2009) offers one explanation of self-stereotyping effects in aging, proposing that negative attitudes about old age and aging–learned early in life–become self-relevant later in life, when one identifies as old, and increase in salience in response to social and environmental cues. However, stereotype embodiment is an evolving process as there is no liminal chronological age that defines when one considers themselves old. Even outside of conscious awareness, negative attitudes can be self-fulfilling in terms of hindered performance through reduced expectations and lower self-efficacy. Further, given that we are not born into “old age,” but instead transition into this group, older adults may be ill-equipped to cope with common and sometimes socially acceptable ageist cues–a problem that might be pronounced for young-old adults (who are new to being “old”) and late middle-aged adults (who are anticipating and perhaps fearing being “old”). While positive stereotype primes could boost performance and negative stereotype primes might hinder performance, one meta-analysis suggests that the impact of negative age primes is much more influential than positive age primes (Meisner, 2012). Another meta-analysis confirmed that negative age primes can impair older adults’ memory performance specifically (*d* = 0.38; Horton et al., 2008). However, instead of internalizing negative old age stereotypes, older adults sometimes respond with age-group dissociation, distancing themselves from the stereotypes (e.g., feeling younger than same-aged peers), perhaps to protect their self-concept (Weiss and Kornadt, 2018).

Different types of stereotype activation effects on older adults’ episodic memory, including age-based stereotype threat and self-stereotyping or priming effects, have been extensively documented using a variety of creative paradigms (for reviews and meta-analyses, see Hess, 2006; Horton et al., 2008; Chasteen et al., 2011; Meisner, 2012; Lamont et al., 2015; Armstrong et al., 2017; Barber and Lui, 2020). Common “threat” paradigms include explaining that the purpose of the study was to compare the performance of older adults to that of younger adults and emphasizing the age-sensitive nature of the test. Researchers may even explicitly state that older adults are not expected to do as well because they are old, may include a younger adult confederate in the testing session (Kang and Chasteen, 2009; Popham and Hess, 2013; Swift et al., 2013; Fernández-Ballesteros et al., 2015; Mazerolle et al., 2017), or may ask participants to read news articles or watch videos describing age-related deficits in memory (Hess et al., 2003; Hess and Hinson, 2006; Thomas and Dubois, 2011; Wong and Gallo, 2018; Marquet et al., 2019). Researchers have also manipulated task instructions to emphasize or de-emphasize the memory component of the testing (Rahhal et al., 2001; Hess et al., 2004; Chasteen et al., 2005; Desrichard and Köpetz, 2005; Sindi et al., 2013; Bouazzaoui et al., 2016). Common “priming” paradigms include embedding stereotype stimuli into other tasks, such as lexical decision tasks (e.g., identifying words versus pronounceable non-words), sentence scramble tasks, or rapid presentation of this stimuli on a computer screen, just above or below participants’ perceptual thresholds (Levy, 1996; Hess et al., 2004; Chasteen et al., 2005; Levy and Leifheit-Limson, 2009; Eibach et al., 2010).

Overall, research using these different paradigms demonstrates poorer performance under negative stereotype conditions than neutral or positive stereotype conditions. Stereotype effects may be more pronounced when the domain of the stereotype matches the performance outcome, such as pairing *senile* and *forgetful* with a memory test (Levy and Leifheit-Limson, 2009). Some meta-analytic work suggests that the characteristics of stereotype manipulations matter greatly. For example, Lamont et al., 2015) found that age-based stereotype threat manipulations that emphasized opinion-based statements, rather than factual ones (e.g., news articles), were more threatening. Armstrong et al., 2017) found that older adults’ episodic memory performance was more sensitive to blatant age-based stereotype manipulations than subtle ones. However, Weiss and Kornadt (2018) argue that blatant age-stereotype activations may be more likely to promote age-dissociation, while the subtle age-stereotype manipulations may promote internalization of the stereotypes. Potentially, stereotype awareness could prompt age group dissociation (although attempts to counteract the stereotype could be cognitively demanding and thus disrupt memory performance further; Pennington et al., 2016).

Stereotype effects may also be moderated by pre-existing individual beliefs about memory and age. Evidence suggests memory self-efficacy and one’s evaluation of their memory at baseline may moderate stereotype effects, and that individuals with low memory self-efficacy and higher dementia worry are the ones most susceptible to stereotype effects in clinical settings (Desrichard and Köpetz, 2005; Fresson et al., 2017; Schlemmer and Desrichard, 2018; but see Chasteen et al., 2005). Stereotype effects may also be moderated by memory achievement, or the value and importance a person places on their memory. For example, Hess et al. (2003) found that the inferior memory performance for participants in a negative stereotype condition–compared to positive and neutral stereotype conditions–was exaggerated as level of memory achievement increased, although they did not replicate this effect in a follow-up study (Hess et al., 2004). Weiss (2018) reported poorer memory following a stereotype manipulation, but only for older adults who initially believed that decline with memory was inevitable. Stereotype threat may also be moderated by individuals’ attitudes toward old age and aging satisfaction, wherein the impact of stereotype threat on memory performance is worse for people who hold more negative age attitudes (Fernández-Ballesteros et al., 2015) and older subjective age may relate to greater sensitivity to stereotype threat effects on memory (Eibach et al., 2010). Alternatively, strong group identification with “old” may buffer against age-based stereotype threat effects (Kang and Chasteen, 2009).

Related research suggests that personal beliefs about memory and age are also sensitive to stereotype effect manipulations. For example, Hess and Hinson (2006) found that a stereotype manipulation impacted several metamemory beliefs, such as a sense of personal control over one’s memory performance. In that research, changes in memory beliefs were a more important predictor of memory performance than the stereotype manipulation itself (Bouazzaoui et al., 2016) demonstrated that older adults assigned to a stereotype threat condition (exposed to negative age stereotypes in a questionnaire) reported more subjective memory complaints and lower memory self-efficacy than a no-threat comparison group. Further, they found that the post-manipulation memory complaints and memory self-efficacy fully mediated the effect of the stereotype manipulation on memory performance. Beliefs about age may also be affected by stereotype manipulations, although the mixed results sometimes evidence assimilation effects (self-beliefs aligned with the stereotypes) and other times they represent age-dissociation effects. For example, after reading negative age stereotypes presented in a fake news article (such as in a traditional age-based stereotype threat manipulation), healthy middle-aged and older adults reported feeling older (Kotter-Grühn and Hess, 2012). In contrast, older adults primed with loss-oriented negative age stereotypes in an “Aging Quiz,” compared to those completing the quiz with growth-oriented positive age stereotypes or neutral age stereotypes, felt younger, reported weaker identification with the “old age” group (Weiss and Lang, 2012), and rated themselves as being more dissimilar to pictures of older people (Weiss and Freund, 2012). Notably, many studies have established links between personal memory and age beliefs and subjective and objective cognitive performance (Beaudoin and Desrichard, 2011; Lachman and Agrigoroaci, 2012; Stephan et al., 2014, 2017). Thus, these findings underscore the value of assessing personal beliefs following stereotype manipulations.

Some controversy surrounds whether stereotype priming manipulations create stereotype threat, or which specific paradigms represent “true” age-based stereotype threat (Chasteen et al., 2005; Kang and Chasteen, 2009), and stereotype threat and stereotype priming effects may occur concurrently (Wheeler and Petty, 2001). A few studies have combined explicit threat-type manipulations with implicit priming-type manipulations (e.g., Hess et al., 2004; Bouazzaoui et al., 2016). Threat appraisal or perceived stereotype threat is often not assessed in studies of stereotype effects on memory. However, when perceived threat is measured, it generally does not differ between the stereotype groups and comparison groups as expected (but see Swift et al., 2013), but it is age-sensitive, negatively related to memory performance, and positively related to anxiety (Chasteen et al., 2005; Kang and Chasteen, 2009; Popham and Hess, 2013; Marquet et al., 2019). Chasteen et al., 2005) reported that perceived stereotype threat fully mediated the effect of age on memory performance, and later found that perceived stereotype threat moderated their stereotype manipulation, wherein memory performance was poorer for those in the stereotype group who also reported higher perceived stereotype threat (Kang and Chasteen, 2009). The variance in reported levels of perceived stereotype threat unrelated to experimental manipulations suggests that some older adults may feel threatened from other characteristics of the testing situation, separate from the stereotype manipulations. Often these studies are conducted in the laboratory or clinic with abstract memory tasks (but see Sindi et al., 2013). As such, they might not generalize to the “real world,” especially given the social and motivational impacts on memory in aging as described above. Concern regarding the practical everyday impact of stereotype threat is not specific to age-based stereotype threat effects, as it has been voiced as well by scholars of gender-based stereotype threat (Stoet and Geary, 2012; Flore and Wicherts, 2015).

The present study aimed to increase our understanding of the potential “real world” impact of negative age and memory stereotypes on middle-aged and older adult’s episodic memory performance. Using a between-groups design, participants completed a story recall memory test after completing tasks with or without negative age and memory stereotype stimuli. The research was designed to be naturalistic and ecologically valid in two primary ways. First, familiar newspaper-like materials were used: Age and memory stereotype stimuli were subtly presented in comics and puzzles, and the memory performance was assessed via story recall, a meaningful, everyday memory task like retelling a news article. Second, participants were tested in familiar settings at preferred times. Testing occurred in participants’ homes and community meeting rooms, rather than a university research laboratory or clinic. Participants were able to schedule testing sessions at the times and days (including weekends) of their choice. Additionally, some participants were recruited from the extended social networks of research assistants (e.g., family friends and neighbors, shared religious and other community groups), which potentially could have encouraged a sense of personal connection to the research. In addition to better representing “real world” experiences, these testing characteristics might also be less stressful for late middle-aged and older adults (Sindi et al., 2013).

The primary purpose of the study was to examine whether this naturalistic design would replicate established stereotype effects (Horton et al., 2008; Lamont et al., 2015; Armstrong et al., 2017; Barber, 2020). Specifically, we expected that episodic memory performance would be poorer for participants exposed to negative old age and memory stereotype stimuli (stereotype group), compared to those exposed to neutral stimuli (control group). We also aimed to explore potential mediators and moderators of the stereotype effects. Given meta-analyses suggesting that subtle stereotype primes may be more disruptive to episodic memory than blatant ones, along with the results of studies that manipulated awareness of age stereotype presentations (Hess et al., 2004; Lamont et al., 2015; Armstrong et al., 2017), we also expected the stereotype effect to be moderated by awareness of the stereotype stimuli. Specifically, we expected better memory performance for participants reporting awareness of the stereotyped stimuli, compared to participants who were unaware, with aware participants possibly showing stereotype lift effects.

Given the relative lack of stereotype research including explicit comparisons of late middle-aged adults and older adults for memory, it is not clear whether these two groups would react in the same way. Some past research has shown that young-old adults (e.g., in the 60’s) may be most vulnerable to impairments related to age stereotype presentations, whereas early middle-aged adults and older-old adults (e.g., those over 70 years old) may be resilient (Hess et al., 2004; Eich et al., 2014). In this study, both groups might self-stereotype and show reductions in performance after exposure to naturalistic negative age and memory stimuli Alternatively, stereotype lift might be evident for the middle-aged group and not the older group, or for the least threatened among both age groups.

To address important issues from past literature, we explored whether reactions to stereotyped stimuli were moderated by pre-existing beliefs about age and memory. We also aimed to determine whether the post-test effects of the “real world” stereotype manipulation would be consistent with age-based stereotype threat theory (Steele, 1997; Shapiro and Neuberg, 2007; Lamont et al., 2015) and/or with stereotype priming effects as in stereotype embodiment theory (Horton et al., 2008; Levy, 2009). If the naturalistic stereotype presentation activated an age-based stereotype threat effect, then we would expect evidence of hot cognitive changes (Wheeler and Petty, 2001), specifically the stereotype group to report greater perceived stereotype threat (Chasteen et al., 2005; Kang and Chasteen, 2009; Swift et al., 2013) and higher levels of state anxiety (Osborne, 2001; Swift et al., 2013; but see Hess et al., 2003, 2004, 2009; Hess and Hinson, 2006) than the control group, and these effects could mediate the impact of the stereotype manipulation on memory performance. If the stereotype manipulation acted more like stereotype priming, then participants might respond with self-stereotyping as in stereotype embodiment, or they might demonstrate age-group dissociation. In the case of self-stereotyping, the stereotype-exposed group might report worse memory evaluations (Bouazzaoui et al., 2016; Chasteen et al., 2005; Hess and Hinson, 2006; Wong and Gallo, 2018) and older subjective ages (Hess et al., 2003; Kang and Chasteen, 2009; Eibach et al., 2010; Kotter-Grühn and Hess, 2012; Fernández-Ballesteros et al., 2015; Marquet et al., 2019) than the control group, and these effects could also operate as mediators. Alternatively, reports of better memory evaluation and younger subjective ages for the stereotype group than the control group might represent an age group-dissociation.

## Materials and Methods

### Participants

A convenience sample of community-dwelling adults were recruited primarily from word-of-mouth (e.g., family friends, community groups), as well as from existing university participant pools and advertisements (e.g., posted to Nextdoor app). Participants were compensated with their choice of $10 or a paper packet of compiled memory exercises and information about memory. Eligible participants were at least 50 years old (range: 50–88 years old; *M*_age_ = 63.35 years; *SD*_age_ = 9.92 years), reported hearing adequate to complete a telephone interview, and rated their overall English ability greater than or equal to 6 on a scale from 1 = *poor* to 10 = *excellent*. Participants were classified as late middle-aged (50–64 years old) or older (65+ years old).

Research assistants confirmed participant eligibility in a preliminary phone interview. In addition to general demographics and questions about health, participants answered questions about beliefs, specifically personal control beliefs (Lachman and Weaver, 1998) and aging satisfaction (Lawton, 1975). Cognitive tests administered by phone included assessments of working memory (backward digit span; Wechsler, 1997) and immediate and delayed recall of a word list (Lezak, 1995) from the Brief Test of Adult Cognition by Telephone (BTACT); these measures are reliable and valid when administered by phone to middle-aged and older adults (Lachman et al., 2014). Participants were free from cognitive impairment. Research assistants administered the Telephone Interview for Cognitive Status (TICS) (Brandt et al., 1988) to the participants who recalled fewer than five of fifteen words on the immediate recall task (Lezak, 1995; Lachman et al., 2014) and had difficulty following instructions during the phone interview. Four participants completed the TICS, and all scored above 31, the recommended cutoff score.

This manuscript reports data from 51 participants who completed both the phone interview and the in-person assessment. Sensitivity analyses in G^∗^Power (version 3.1.9.4; Faul et al., 2007) indicated that a sample size of 51 participants would have 80% power (1–β error probability) to detect a large effect size of Cohen’s *f* = 0.40 with α = 0.05 in a two-groups analysis of covariance (ANCOVA) with one covariate (the analysis for our primary research aim). Meta-analyses of stereotype activation effects have reported effect sizes of *d* = 0.34–0.38 (Walton and Cohen, 2003; Horton et al., 2008; Lamont et al., 2015). One additional participant was excluded for inability to follow directions during the in-person assessment. Overall, participants were healthy (*M* = 8.00, *SD* = 1.61, rated on a scale from 1 = *poor* to 10 = *excellent*) and well-educated (*M*_y__ea__rs_ = 15.10, *SD*_y__ea__rs_ = 2.79). Table 1 reports descriptive data by age group and experimental condition for health, cognition, beliefs, and basic demographic information.

**TABLE 1:** Demographics and background information by experimental condition and age group.

	Stereotype	Control	Total
	*M* (*SD*)	*M* (*SD*)	*M* (*SD*)
**Sample size (*n*)**	26	25	51
Late middle-aged adults	15	16	31
Older adults	11	9	20
**Gender (% female)**	65%	68%	67%
Late middle-aged adults	67%	63%	64%
Older adults	64%	78%	70%
**Retirement status (% retired)**	35%	20%	28%
Late middle-aged adults	20%	6%	13%
Older adults	55%	44%	50%
**Age (years)**	64.58 (9.73)	61.16 (9.93)	62.90 (9.88)
Late middle-aged adults	57.20 (4.44)	54.81 (3.71)	55.97 (4.19)
Older adults	74.64 (4.03)	72.44 (6.78)	73.65 (5.40)
**Years of education**	16.04 (2.85)	16.16 (2.78)	16.10 (2.79)
Late middle-aged adults	16.47 (2.75)	16.31 (2.33)	16.39 (2.50)
Older adults	15.45 (3.01)	15.89 (3.59)	15.65 (3.20)
**Physical health rating (1 = *poor* to 10 = *excellent*)**	8.23 (1.51)	7.76 (1.72)	8.00 (1.61)
Late middle-aged adults	8.27 (1.58)	7.88 (1.71)	8.06 (1.63)
Older adults	8.18 (1.47)	7.56 (1.81)	7.90 (1.62)
**Vision rating (1 = *poor* to 10 = *excellent*)**	7.77 (1.42)	7.32 (1.80)	7.55 (1.62)
Late middle-aged adults	7.87 (1.19)	7.06 (1.81)	7.45 (1.57)
Older adults	7.64 (1.75)	7.78 (1.79)	7.70 (1.72)
**Hearing rating (1 = *poor* to 10 = *excellent*)^†^**	8.04 (1.93)	8.16 (1.60)	8.10 (1.76)
Late middle-aged adults	8.27 (2.09)	8.63 (1.03)	8.45 (1.61)
Older adults	7.73 (1.74)	7.33 (2.12)	7.55 (1.88)
**Working memory (backward digit span; 0–7)^†^**	4.73 (1.37)	4.76 (1.39)	4.75 (1.37)
Late middle-aged adults	5.20 (1.08)	4.81 (1.28)	5.00 (1.18)
Older adults	4.09 (1.51)	4.67 (1.66)	4.35 (1.57)
**Immediate recall (RAVLT; 0–15 words)**	7.54 (2.98)	7.17 (2.75)	7.36 (2.85)
Late middle-aged adults	7.47 (2.83)	6.87 (2.72)	7.17 (2.74)
Older adults	7.64 (3.33)	7.67 (2.87)	7.65 (3.05)
**Delayed recall (RAVLT; 0–15 words)**	5.42 (2.60)	5.33 (2.24)	5.38 (2.41)
Late middle-aged adults	5.87 (2.33)	5.20 (2.34)	5.53 (2.32)
Older adults	4.82 (2.93)	5.56 (2.19)	5.15 (2.58)
Perceived mastery (1–6)*	5.08 (0.64)	5.25 (0.60)	5.16 (0.62)
Late middle-aged adults	5.27 (0.63)	5.38 (0.39)	5.32 (0.51)
Older adults	4.82 (0.60)	5.02 (0.85)	4.91 (0.71)

*Age difference, *p* < 0.05. ^†^Age difference, *p* < 0.10. No condition differences significant. RAVLT, Rey Auditory Verbal Learning Test.

To test for baseline differences between groups randomly assigned to the stereotype condition or the control condition, we conducted a series of independent samples *t* tests for each of the continuous variables reported in Table 1 (e.g., years of education, working memory scores). None of these differences were significant, nor trended toward significance, all *p*s > 0.100. We also found no significant difference in the proportions of participants in the stereotype condition and in the control condition who were female, χ^2^ = 0.04, *p* = 0.843, or who were retired, χ^2^(1,51) = 1.37, *p* = 0.242. This pattern of results suggests that random assignment to condition was successful in creating comparable groups.

We also conducted independent samples *t* tests to compare participants assigned to the two age categories. The results suggested, on average, a greater sense of perceived mastery (global personal control beliefs) for late middle-aged participants than older participants. The mean difference of 0.41, 95% CI [0.07, 0.75], was significant, *t*(49) = 2.42, *p* = 0.019, and represented a large effect, *d* = 0.80, consistent with other research (Robinson and Lachman, 2017). Results also suggested marginally significant effects for hearing and working memory, consistent with typical age-related changes (Li-Korotky, 2012; McCabe and Loaiza, 2012) with late middle-aged participants reporting better hearing, *M*_diff_ = 0.90, 95% CI [−0.09,1.89], *t*(49) = 1.82, *p* = 0.073, *d* = 0.56, and scoring higher on the backward digit span task, *M*_diff_ = 0.65, 95% CI [−0.13, 1.43], *t*(49) = 1.69, *p* = 0.098, *d* = 0.55. No other baseline differences between late middle-aged and older participants were significant, *p*s > 0.100.

### Procedures

Participants completed a 15-min preliminary phone interview and a 45- to 60-min in-person assessment. Most participants completed their in-person assessment in the same week as their phone interview. Research assistants followed detailed protocols for administering both interviews, which were audio recorded for quality control. The order of presentation of tests and measures for the phone interview and in-person assessment are summarized in Table 2 along with relevant reliability statistics and citations. All study procedures and materials were approved by the University of Florida Institutional Review Board 02 (#2015-U-0680).

**TABLE 2:** Presentation order of telephone interview and in-person assessment tests and measures.

Tests and Measures	Cronbach’s α	Citation
**Phone Interview**		
Backward Digit Span	–	Lachman et al., 2014
Rey Auditory Verbal Learning Test–Immediate Recall	–	Lezak, 1995
Category Fluency Test	–	Lachman et al., 2014
Health and Demographics Questionnaire	–	Strickland-Hughes et al., 2016
Rey Auditory Verbal Learning Test–Delayed Recall	–	Lachman et al., 2014
Perceived Mastery^a^	0.70	Lachman and Weaver, 1998
Attitudes Toward Aging Subscale of Philadelphia Geriatric Center Morale Scale^a^	0.81	Lawton, 1975
Telephone Interview for Cognitive Status*	–	Brandt et al., 1988
**In-Person Assessment**		
Outcome Expectancies Questionnaire	–	(created for present study)
MIA Achievement^b^	0.76	Dixon et al., 1988
MIA Anxiety^b^	0.83	Dixon et al., 1988
MIA Capacity^b^	0.86	Dixon et al., 1988
MIA Control^b^	0.71	Dixon et al., 1988
Stereotype Manipulation	–	
Comics Rating	–	(created for present study)
Word Search	–	(created for present study)
Word Jumbles	–	(created for present study)
Story Recall	–	Dixon et al., 1989
State Anxiety	0.93	Abrams et al., 2006
Subjective Age Identity	0.96	Strickland-Hughes et al., 2016
General Memory Evaluation	0.86	West et al., 2003
Perceived Stereotype Threat	0.79	Chasteen et al., 2005
Health and Medications Questionnaire	–	(created for present study)
Stereotype Stimuli Awareness Manipulation Check	–	(created for present study)

*Only administered to some participants based on ability to follow directions and immediate recall scores. ^*a,b*^Indicates surveys presented with items together in randomized order. Cronbach’s α scores reported are from the cited research; internal consistency scores from the present data are reported in text. The phone cognitive tests have strong test–retest reliability and convergent validity, as well as moderate correlations between phone and in-person administration with older adult samples (Lachman et al., 2014). MIA, Metamemory in Adulthood.

Participants completed the in-person assessments in small groups with no more than four participants. Because the experimental stimuli (described below) were presented in paper-and-pencil packets, small groups often included participants randomly assigned to both conditions (stereotype and control). Importantly, in-person interviews were not conducted in an on-campus psychology laboratory setting, which itself may induce stereotype threat (Barber and Lui, 2020). Rather, the in-person interviews took place in participants’ homes and familiar community meeting areas in Georgia and Florida that were quiet and free from distractions.

For the in-person interview, instructions were presented orally and on paper packets on which participants wrote their responses. Informed consent was followed by general instructions and information about the story recall task. Baseline memory beliefs (as potential moderators of stereotype effects) were then assessed prior to completion of the three naturalistic activities that either presented stereotypic age and memory stimuli or control stimuli: rating comics, completing a word search, and solving a word jumble. Following the stereotype manipulation, participants completed the story recall task and surveys on their state anxiety and perceived stereotype threat (to identify if the manipulation functioned like an age-based stereotype threat manipulation) and on their subjective age and memory evaluation (to identify if the stereotype presentation resulted in assimilation effects consistent with stereotype embodiment theory or in age-group dissociation effects). The interview concluded with a manipulation check and debriefing. Each of these specific measures are described below (section “Measures”).

### Measures

#### Naturalistic Stereotype Activation

The independent variable in this research was experimental condition: Participants were randomly assigned to complete naturalistic newspaper-style activities (word search, word jumble, and rating comics) with or without embedded words that primed negative age and memory stereotypes (e.g., forgetful, weak). The comics for the stereotype condition included punchlines related to old age and/or memory, whereas the control comics included punchlines related to other age groups and other cognitive processes. To select the word stimuli for the stereotype and control versions of the word search and word jumble activities, we aggregated words used in past age-based stereotype threat and stereotype priming research (Bargh et al., 1996; Levy, 1996; Chasteen et al., 2002; Hess et al., 2004), as well as designated old-age traits from aging attitudes research (Schmidt and Boland, 1986; Hummert et al., 1994; Schwartz and Simmons, 2001; Grühn et al., 2011). Stereotypical words were selected and categorized as relating primarily to “old age” (e.g., *frail*, *helpless*, *lonely*) or “memory” (e.g., *senile*, *misplace*, *impaired*). These words were then pseudo-randomly assigned to lists for the activities (word search, word jumble, and examples for each) without replacement, so that each word could only be included in one item. For all three activities, two alternative versions were used for both the stereotype condition and the control condition, with the versions assigned by counterbalancing, to minimize stimulus-specific effects. The three activities are described in detail below.

##### Comics rating

For the comics rating activity, participants used an 11-point scale to rate the punchline (0 = *not at all funny* to 10 = *very funny*), the extent to which each comic “shows real life” (0 = *very untrue* to 10 = *very true*), and the tone of each comic (0 = *very mean* to 10 = *very nice*). Each participant had to rate six total comics, including three neutral ones. The neutral comics were negatively valanced but absent of any themes related to age or cognition. For example, one neutral comic was a *Robots Read News* cartoon by Scott Adams where a robot newscaster says humans are awful “when they are awake.” All participants in both experimental conditions rated the three neutral comics, which permitted examination of potential response biases between the two conditions. For the stereotype condition, participants rated a comic related to old age, a comic related to memory, and a comic related to both old age and memory. For example, one of the old age and memory comics was a *Pickles* cartoon by Brian Crane that showed an older man misplacing his glasses, which were on his head. For the control condition, participants rated a comic related to age (but not old age), cognition (but not memory), and age and cognition (again, neither related to old age or memory). For example, one of the age and cognition comics was a *Zits* cartoon by Jerry Scott showing a teenager struggling with attention (his history text “went in one eye and out the other”).

Comics were identified and assigned to the alternative versions of the stereotype condition and control condition tasks based on a multi-stage rating process. First, research assistants curated a selection of 85 comics with themes related to age or cognition from current newspapers and online archives. Research assistants and their friends and family members rated these comics on 11-point scales for negativity (e.g., pleasant-unpleasant), realism (e.g., accurate-inaccurate), relatability (e.g., just like my life-nothing like my life), humor (e.g., not at all funny-very funny), annoyance (e.g., made me feel not at all bothered-extremely bothered), and age- and memory-salience. Based on these ratings, 15 comics were selected for additional review by an independent sample of middle-aged and older adults (*N* = 34) using an online survey. The final comics selected for the research were similar in terms of overall negativity, realism, relatability, humor, and annoyance, as rated by that independent sample. Further, comics were matched for size and format across conditions (e.g., number of panes; horizontal arrangement).

##### Word search

Participants received instructions on how to solve the word search and were given a laminated reference sheet with additional “tips and tricks” (reference sheet included in Supplementary Materials). To enhance their engagement with the stimulus words, participants were instructed to cross off words, once located, to help track progress. To ensure that the instructions were clear, participants practiced by completing a 6-by-6 letter grid sample word search that included three words to find (printed in a box at the bottom of the page). The main word search was a 10-by-10 letter grid with nine words to find (also printed at the bottom of the page). Participants had 3 min to solve the word search. In the control condition, all words for the sample and main word search were neutral and unrelated to old age or memory. In the stereotype condition, the words in the sample word search (*aged*, *recall*, and *watch*) included category age and memory terms to cognitively prime these categories during the subsequent task (such as in Hess et al., 2004). The main word search in the stereotype condition included three words related to memory, three words related to old age, and three neutral words, unrelated to age or memory. For the word search puzzles, the final four lists (two stereotype and two control sets of stimuli) were comparable in terms of mean word length (in letters) and the proportions of words of each length (ranging from four to eight letters long). All word search puzzles are included in Supplementary Materials.

Performance on the word search was not of central interest in this study. Rather, we designed the activity to maximize exposure to the stimuli. However, we noted that all participants found all three of the words in the sample, and half of the participants (51%) found all nine of the words in the main word search.

##### Word jumbles

The final activity in the naturalistic stereotype activation was completion of a word jumble. A word jumble has multiple components, which must be used to create a final word or phrase (often a pun) that fits a cartoon and its accompanying descriptor. To solve any jumble, participants first unscramble the letters for common words. In the solution box for each of those common words, some letters are circled. The circled letters can then be re-arranged to write the required final word or phrase. A list of words including correct answers and distractors were printed at the bottom of the page. This procedure deviated from the standard newspaper format but ensured exposure to the stimuli, even if participants did not solve the jumbles, and made the task easier. As with the word search, participants received instructions and completed an example before completing the main jumble. Participants were again encouraged to use a laminated reference sheet with “tips and tricks” (see Supplementary Materials).

We created the jumbles by replacing words from real newspaper jumbles with our stimuli. The cartoons and the final correct responses were unchanged. The example was the same for all participants and included two four-letter scrambled words with four possible solutions at the bottom. Participants were given 5 min to complete the main jumble. All four versions of the main jumble (two stereotype condition and two control condition) used five scrambled common words (one 5-letters long, three six-letters long, and one eight-letters long), and showed ten words printed at the bottom of the page. The final two-word phrase required for each cartoon used eight to ten circled letters. For the stereotype condition, three of the five scrambled words and three additional words included as distractors were taken from the old age and/or memory stimuli lists described above. All words in the control condition were neutral, unrelated to old age nor memory.

Again, performance on this task was not important, although we encouraged all participants to solve the jumble. Indeed, 86% of participants correctly unscrambled all five words in the jumble.

#### Story Recall Test

We assessed episodic memory, our primary outcome measure, with performance on a story recall task. This everyday memory task was selected for its fit with the naturalistic stereotype stimuli, given the task’s similarity to reading and recollecting a newspaper article. Participants were given 1 min to encode the story. Two matched eight-sentence stories were counterbalanced by experimental condition [from Dixon et al. (1989) complication of structurally equivalent texts]. One story was about an older couple camping during the summer, and the other story was about a couple expecting the birth of a grandchild. Four minutes were allotted for recall, when participants were instructed to: *Please write down everything that you remember from the story. You need to recall the story as precisely as possible*. Story recall was calculated as the percent of words recalled from the story text, following procedures commonly used in our laboratory (Smith, 2014; West et al., 2018).

#### Stereotype Stimuli Awareness

A manipulation check was conducted at the end of the in-person assessment. All participants were asked whether they noticed anything “in particular” about the (a) comics, (b) word search, and (c) word jumble. If they indicated “yes,” they were asked to describe what they noticed. Two independent raters evaluated each response and coded whether participants in the stereotype condition indicated being aware of the old age and/or memory theme(s) in the activities. Disagreements in coding were rare and were resolved through discussion.

#### Indicators of Age-Based Stereotype Threat and Stereotype Priming Effects

We assessed four measures following the stereotype manipulation and memory testing to assess whether responses reflected age-based stereotype threat (higher perceived stereotype threat and higher state anxiety) and/or stereotype embodiment (lower general memory evaluation and older subjective age). Responses to these measures for participants in the stereotype group may reflect their reaction to the stereotype stimuli and memory testing, whereas responses from the control group reflect reactions to the testing itself.

##### Perceived stereotype threat

Participants responded to four items about judgments of their personal memory ability from the Perceived Stereotype Threat measure (Chasteen et al., 2005) following the story recall task. An example item is *I often feel I have to prove to others that their perceptions of my memory ability are wrong*. Items were rated from 1 = *strongly disagree* to 5 = *strongly agree*. Responses were averaged to calculate a composite score (α = 0.70). Higher scores indicated a greater overall feeling of age-based stereotype threat for memory.

##### State anxiety

State anxiety was assessed with an eight-item self-report survey (Osborne, 2001; Abrams et al., 2006). Participants used a scale ranging from 1 = *not at all* to 7 = *very* much to rate the extent to which they felt under pressure, tense, nervous/jittery, confident, uneasy, calm, afraid of not doing well, and uncomfortable during the story recall task. A state anxiety score was calculated as the mean response to the eight items (α = 0.83) that ranged from one to seven. Responses were reverse coded so that higher scores represented greater level of anxiety.

##### General memory evaluation

The General Memory Evaluation survey (West et al., 2003, 2008) was administered following the stereotype manipulation and story recall task. Responses to three items on a seven-point Likert-type scale (e.g., 1 = *very unsatisfied* to 7 = *very satisfied*) were averaged to calculate an index score (α = 0.73; theoretical range: 1–7). The items concern evaluation of one’s recent memory performance, comparison of one’s memory to that of same-age peers, and overall satisfaction with recent memory performance. Higher values indicate higher perceived recent memory ability.

##### Subjective age identity

Subjective age identity reflects how old people feel (Montepare, 2009). Participants responded to five subjective age identity questions assessing their felt age (e.g., *At this moment, how old do you feel?*) by providing ages in years (Strickland-Hughes et al., 2016). Following standard procedures (Rubin and Berntsen, 2006), we averaged the responses to these five questions to compute a subjective age score (α = 0.83). Then, we calculated proportional subjective age identity scores by dividing the difference between participant’s subjective age and chronological age by their chronological age. Proportional subjective age scores are interpretable as the percentage older (positive scores) or younger (negative scores) one feels, relative to their “actual” age.

#### Baseline Beliefs About Memory and Age

We assessed some personal beliefs about memory aging prior to the stereotype manipulation and the memory testing, consistent with past research (e.g., Hess et al., 2003, 2004). Each of these measures represent general beliefs based on long-term experiences, so we assessed them before the stereotype manipulation as potential moderators.

##### Metamemory in adulthood

We administered four subscales of the Metamemory in Adulthood questionnaire (MIA; Dixon et al., 1988), a reliable and valid survey assessing beliefs about memory. A five-point response scale from *agree strongly* to *disagree* strongly was used for all items. Many items were reverse coded. Subscale scores for MIA Achievement (motivation to perform well; α = 0.73), MIA Anxiety (impact of stress on one’s memory; α = 0.80), MIA Capacity (confidence in one’s ability; α = 0.75), and MIA Control (effect of one’s own effort on memory; α = 0.72) were calculated as means of responses to all items in the subscale, with theoretical ranges from 1 to 5. Higher scores suggest higher levels of those beliefs. For additional survey details, see Dixon et al. (1988).

##### Aging satisfaction

Aging satisfaction was assessed with the Attitudes toward Aging subscale of the Philadelphia Geriatric Center Morale Scale (Lawton, 1975). We averaged responses to eight statements (α = 0.84) about one’s aging experience (e.g., *The older I get*, *the more useless I feel*, and *I have as much pep as I did last year*) that participants made on a six-point Likert-type scale from 1 = *strongly disagree* to 6 = *strongly agree*. Items were reverse coded so that higher scores represented greater satisfaction with one’s aging experience.

## Results

The primary goal of this research was to test whether naturalistic stereotype presentations impacted late middle-aged and older adult’s memory performance. We further aimed to evaluate whether the this expected relationship was moderated or mediated by personal factors (e.g., chronological age, awareness of age stereotypes, beliefs). Additionally, we explored whether the stereotype manipulation impacted perceived stereotype threat, anxiety, memory evaluation, and subjective age, as factors that could provide insight to participants’ responses to the manipulation. Our analytic approach included linear models, e.g., analyses of covariance and multiple linear regressions. For the reported results, all assumptions for the analyses (e.g., no outliers or influential cases, independent errors, homoscedasticity) were met or appropriate techniques or corrections were applied (e.g., weighted least squares regression, bootstrapped confidence intervals). Consistent with other studies (Swift et al., 2013), we included age as a covariate in most analyses in order to test for effects while controlling for the relationship with age, as many of our variables are age-sensitive.

### Impact of Naturalistic Stereotype Manipulation

#### Effect on Story Recall Performance

**FIGURE 1:**
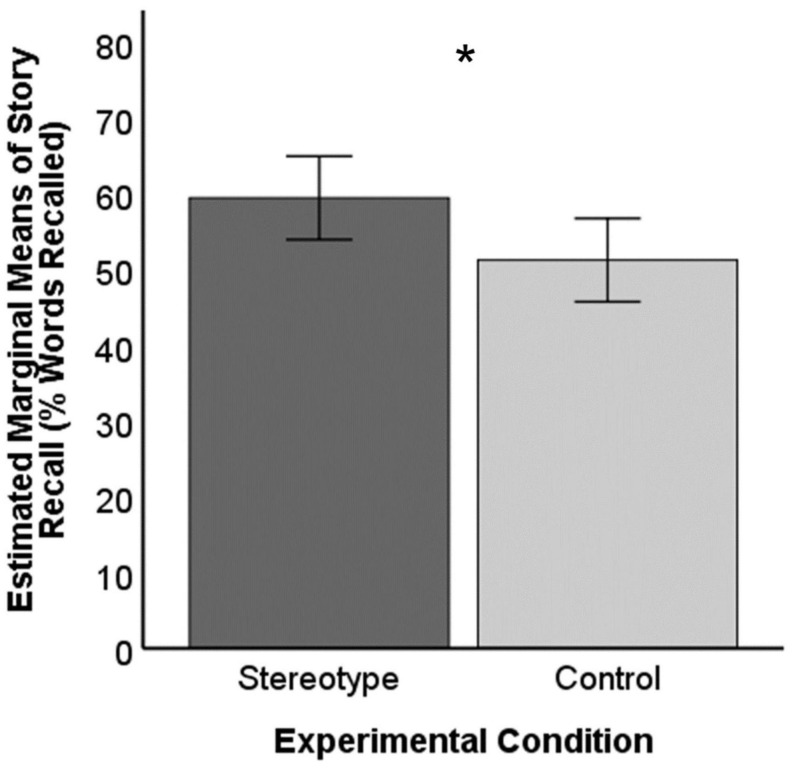
Story recall performance by condition. Error bars represent 95% confidence intervals. Covariate age (in years) evaluated at 63.12. * Indicates significant mean difference, *p* < .05.

To address the primary issue about condition effects, we conducted an ANCOVA to compare mean story recall scores between the stereotype condition and the control condition while accounting for age. We included age as a covariate because chronological age is generally related to episodic memory performance (Park and Festini, 2016; Cabeza et al., 2018). Age was a significant predictor of story recall, *F*(1,47) = 4.17, *p* = 0.047, *r* = 0.22. An increase in 1 year of age was associated with a 0.40% decrement in story recall performance. There was also a significant effect of experimental condition on story recall, *F*(1,47) = 4.43, *p* = 0.041, partial η^2^ = 0.09. Results suggested superior memory performance for the stereotype condition (*EM* = 59.45%, *se* = 2.74%, 95% CI [53.94%, 64.95%]) compared to the control condition (*EM* = 51.25%, *se* = 2.74%, 95% CI [45.74%, 56.76%]). Story recall for participants in the stereotype condition was about 1.16 times that of those in the control condition. This effect is illustrated in Figure 1.

#### Moderation by Age Group

**FIGURE 2:**
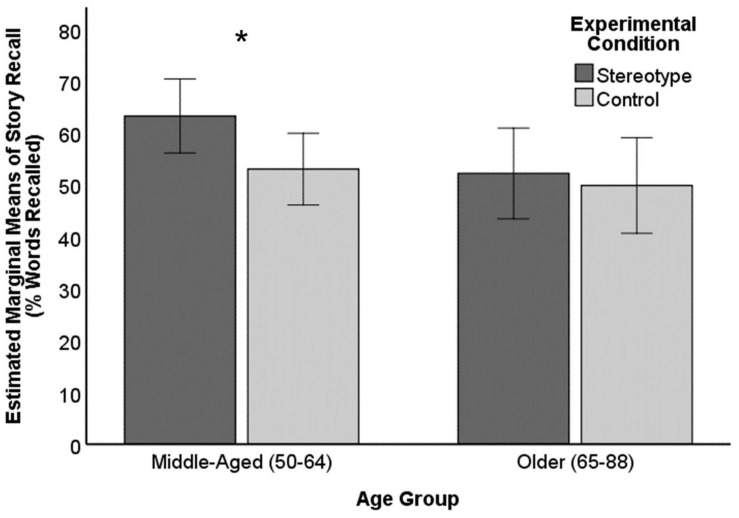
Story recall performance by age group and condition. Error bars represent 95% confidence intervals. * Indicates significant mean difference, *p* < .05.

For our first exploratory question, we tested whether the effect of the stereotype manipulation on memory performance was moderated by age. First, we conducted a 2 condition (stereotype, control) × 2 age group (late middle-aged, older) independent groups analysis of variance (ANOVA) to test whether the impact of the stereotype manipulation was similarly evidenced in both age groups or moderated by age group. The main effect of condition was not significant, *F*(1,46) = 2.46, *p* = 0.125, partial η^2^ = 0.05, although it was significant above, when controlling for an age covariate. The main effect of age group was marginally significant, *F*(1,46) = 3.15, *p* = 0.082, partial η^2^ = 0.082, as late middle-aged participants (*M* = 57.96%, *SD* = 14.94%) trended toward better performance on the story recall task than older participants (*M* = 51.08%, *SD* = 14.33%). The condition × age group interaction effect was not significant, *F*(1,46) = 0.98, *p* = 0.329, partial η^2^ = 0.02. However, as illustrated in Figure 2, pairwise comparisons suggested that for late middle-aged participants, performance was significantly better for the stereotype condition (*M* = 63.24%, *SD* = 12.13%) than the control condition (*M* = 53.01%, *SD* = 15.98%), *M*_diff_ = 10.23, *p* = 0.044, 95% CI [0.31, 20.16]. In contrast, no statistical difference between the stereotype condition (*M* = 52.18%, *SD* = 11.67%) and the control condition (*M* = 49.86%, *SD* = 11.67%) was observed for older participants (*M*_diff_ = 2.33, *p* = 0.714, 95% CI [−15.02, 10.37]) using this more sensitive statistical test.

#### Awareness of Stereotype Stimuli

Of the 26 participants randomly assigned to the stereotype condition, 13 indicated awareness of the age and/or memory themes in the comics and puzzles (“aware” condition), and 13 did not indicate awareness of the themes (“unaware” condition). Even with this small sample size, the results suggested that age was related to awareness of the stereotype stimuli. A marginally significant independent samples *t* test, *t*(24) = 1.93, *p* = 0.065, suggested that those who were aware of the stereotype stimuli were younger (*M* = 61.65, *SD* = 8.61) than those who were unaware of the stereotype stimuli (*M* = 68.55, *SD* = 9.79). The mean difference of 6.98 years, 95% CI [−0.48, 14.44], represented a large effect, *d* = 0.72. It is also important to know whether awareness affected outcomes. Results of an ANCOVA suggested no significant difference in story recall between the “aware” group (*M* = 61.25%, *SD* = 14.09%) and “unaware” groups (*M* = 56.18%, *SD* = 11.59%), while accounting for age, *F*(1,23) = 0.96, *p* = 0.338, partial η^2^ = 0.04.

#### Moderation by Baseline Memory and Age Beliefs

Similarly, we explored whether beliefs about memory (four subscales of the Metamemory in Adulthood questionnaire) and about age (aging satisfaction) moderated the impact of the naturalistic stereotype manipulation on story recall. We conducted a series of moderation analyses using *PROCESS* (version 3.5; Hayes, 2017). None of these beliefs were significant predictors of memory performance, nor were their interactions with condition, based on all *p*s > 0.10.

### Evidence for Stereotype Threat or Stereotype Embodiment

A series of ANCOVAs compared outcomes administered following the stereotype manipulation and memory testing theoretically related to age-based stereotype threat effects (perceived stereotype threat and state anxiety) and stereotype priming effects (memory evaluation and subjective age) as reported by the stereotype and control groups, and accounting for age. For the two outcomes related to stereotype threat, the direction of the differences in the estimated means suggested lower perceived threat (*M*_diff_ = −0.05), lower anxiety (*M*_diff_ = −0.29) for the stereotype condition than the control condition. In terms of possible priming effects, the direction of the differences in the estimated means suggested more positive memory evaluation (*M*_diff_ = 0.35) and older proportional subjective ages (*M*_diff_ = 0.02) for the stereotype condition than the control condition. However, none of these differences were significant, *p*s > 0.100. We did not conduct further tests of mediation for these factors.

**FIGURE 3:**
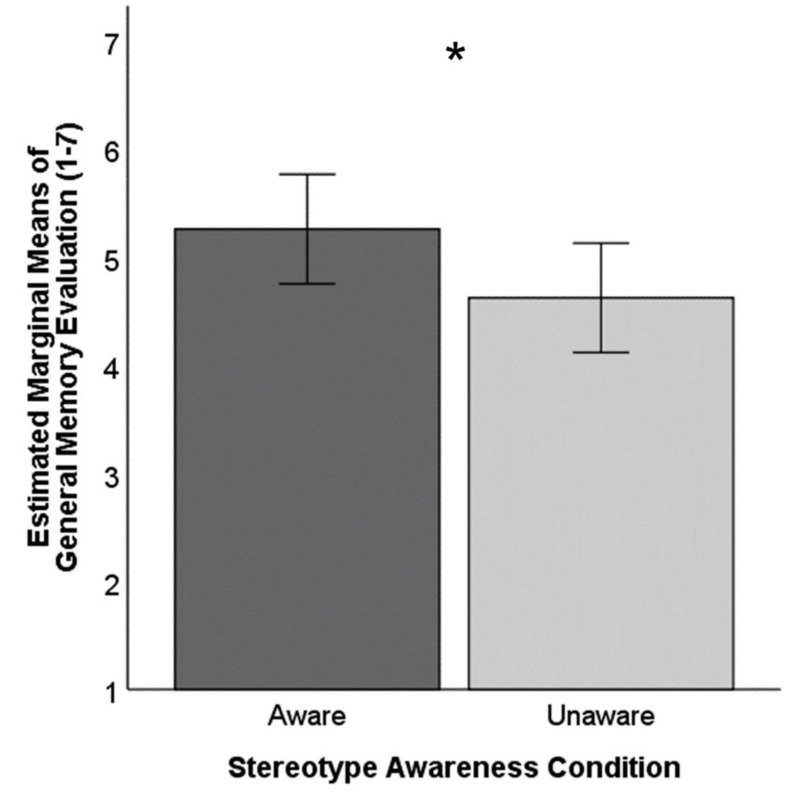
Evaluation of memory ability by stereotype awareness. Error bars represent 95% confidence intervals. Covariate age (in years) evaluated at 65.05. * Indicates significant mean difference, *p* < .05.

We followed these analyses with ANCOVAs comparing these same key outcomes between the “aware” and “unaware” stereotype groups, again accounting for age. Results suggested a trend toward a more positive memory evaluation in the “aware” condition (*EM* = 5.25, *se* = 0.24, 95% CI [4.75, 5.76]) than the “unaware” condition (*EM* = 4.62, *se* = 0.24, 95% CI [4.11, 5.12]), when controlling for age, *F*(1,23) = 3.16, *p* = 0.089, partial η^2^ = 0.12. This effect is illustrated in Figure 3. Age was not a significant predictor of memory evaluation in this model, *F*(1,23) = 0.12, *p* = 0.731. The estimated means for the “aware” condition suggested lower perceived stereotype threat (*M*_diff_ = −0.26) and higher anxiety (*M*_diff_ = 0.36) than the “unaware” condition, but these mean differences were not significant, *p*s > 0.10.

### Moderation by Perceived Stereotype Threat

**FIGURE 4:**
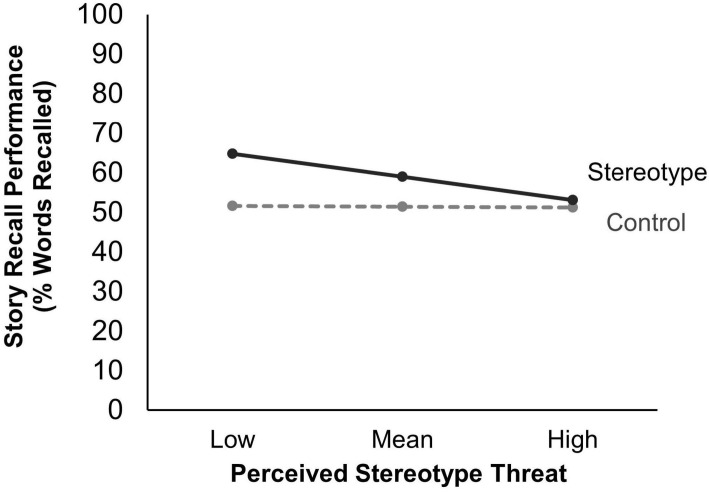
Story recall performance by condition and perceived stereotype threat. Perceived threat is mean centered. Low is defined as –1 *SD* (0.89), and High is defined as +1 *SD* (0.89).

Because of its theoretical importance, we conducted a moderation analysis using *PROCESS* (version 3.5; Hayes, 2017) to test whether perceived stereotype threat (mean centered) moderated the impact of condition on story recall, with age included as a covariate. The overall model explained about 20% of the variance in story recall performance, *F*(4,45) = 4.83, *p* = 0.003, *R*^2^ = 0.20. The main effect of condition was significant (*p* = 0.050), although the main effect of perceived threat (*p* = 0.934) and the condition × perceived threat interaction effect (*p* = 0.130) were not. Given the near marginal significance of the interaction effect, we decomposed the effect to explore the differences in memory performance between the two conditions for varying levels of perceived threat. Story recall performance was higher for the stereotype condition than the control condition when perceived stereotype threat was low, *b* = −13.12, *se* = 4.30, 95% CI [−21.78, −4.45], *t* = −3.05, *p* = 0.004, or average, *b* = −7.52, *se* = 3.74, 95% CI [−15.05, 0.01], *t* = −2.01, *p* = 0.050. However, the difference in story recall performance between the two conditions was not significant when perceived stereotype threat was high, *b* = −1.92, *se* = 5.99, 95% CI [−13.98, 10.13], *t* = −0.32, *p* = 0.749. The Johnson-Neyman method indicated that the upper bound of the zone of significance was −0.001 (56% of the mean-centered perceived threat values were below this boundary). Results of the moderation analysis are summarized in Table 3 and illustrated in Figure 4.

**TABLE 3:** Linear model of condition and perceived threat predicting memory performance.

	*b* [95% CI]	*SE B*	*t*	*p*
Constant	70.89 [48.31, 93.46]	11.21	6.33	<0.001
Condition	7.52 [−0.01,15.05]	3.74	2.01	0.050
Perceived threat (centered)	−0.25 [−6.71, 6.21]	3.21	−0.08	0.934
Condition × Perceived threat	6.30 [−14.53, 1.93]	4.09	−1.54	0.130
Age (centered)	−0.31 [−0.64, 0.02]	0.16	−1.89	0.065

*R^2^ = 0.20, *F*(4,45) = 4.82, *p* = 0.003. Condition: 0 = Control, 1 = Stereotype.*

## Discussion

Impaired episodic memory performance related to negative age stereotype priming and age-based stereotype threat are generally, although not exclusively, evidenced in unfamiliar testing environments (e.g., *Memory*, *Aging*, and *Dementia Lab* at Research Intensive Medical University) and with laboratory-type abstract memory tests that might not be important to older adults (Hess, 2014). This research aimed to test whether a naturalistic stereotype manipulation in a supportive testing environment disrupted the memory performance of late middle-aged and older adults, as past research on stereotype threat and stereotype priming has robustly evidenced for older adults (Horton et al., 2008; Lamont et al., 2015; Armstrong et al., 2017; Barber and Lui, 2020). Crucially, late middle-aged is a time of transition, when individuals begin to anticipate moving into the “old” category, with age 65 as a typical cut-off point for recategorization.

Our results provided evidence for rejection of the stereotypes embedded in naturalistic materials. In general, late middle-aged and older adults exposed to the negative age and memory stereotype stimuli performed better on the story recall test than a comparison group exposed to neutral stimuli. Perhaps, when old age stereotypes are presented in favorable conditions (e.g., testing at home at preferred times of day) with familiar, naturalistic tasks, they are “unthreatening,” or the supportive environment and naturalistic materials may promote the requisite motivational arousal and persistent effort to reject behavioral assimilation to the old age categorization (Miron and Brehm, 2006). These testing characteristics may be more supportive for late middle-aged and older participants compared to standard procedures in memory and aging studies (Barber and Lui, 2020). Certainly, the present research did not provide evidence for the stereotype manipulation being “threatening” in ways consistent with an age-based stereotype threat effect. The stereotype group and control group reported comparable levels of perceived stereotype threat and task-related anxiety, suggesting the combination of presenting negative stereotype stimuli with memory testing was not more threatening than memory testing alone. Even though the power analysis suggested that this sample size was appropriate, a larger sample size might have detected such effects. It is important to note that other authors have similar findings. Although some age-based stereotype threat paradigms (e.g., emphasizing that older people are expected to perform worse than younger people) report greater perceived threat and higher anxiety following threat inductions, as compared to neutral or positive conditions (Swift et al., 2013), our finding is consistent with research demonstrating that presentation of age stereotype stimuli in a lexical decision task did not relate to greater perception of age-based stereotype threat (Chasteen et al., 2005) and studies failing to demonstrate a relationship between stereotype manipulations and anxiety (Hess et al., 2003, 2004, 2009; Hess and Hinson, 2006).

Favorable testing conditions for memory performance may be critical here (Hehman and Bugental, 2013). While they did not present stereotype stimuli, Sindi et al., 2012) demonstrated that older adults had better episodic memory performance and less stress (assessed with cortisol levels) when tested in “old favoring” conditions similar to those in our study (e.g., testing during the morning in a familiar environment with socially relevant stimuli and de-emphasizing memory), as opposed to “young favoring” conditions (e.g., testing during the afternoon in an unfamiliar environment with a word list test emphasizing memory). Furthermore, our testing situation may have been “favorable” because most participants experienced “success” completing a cognitive task prior to the memory test. That is, because we wanted to maximize participants’ exposure to the stereotype stimuli in the word search and jumble, we arranged those tasks so that most people would be able to complete both puzzles (e.g., we provided “tips and tricks” sheets and word banks). Inadvertently, the resultant “success” with the puzzles may have promoted memory performance, consistent with findings that older adults show better memory when tests are immediately preceded by successful completion of another cognitive task (Geraci and Miller, 2013; Geraci et al., 2016).

Relatedly, our data suggested that stereotype lift was evidenced for participants reporting average or below average levels of perceived stereotype threat, but not for those with above average levels of perceived stereotype threat. This finding suggests that lower perception of perceived stereotype threat (as might be promoted in favorable testing conditions) could “set the stage” for rejection of negative age and memory stereotypes. We recommend that future research directly compare the naturalistic negative stereotype presentation to presentation of control stimuli in favorable and unfavorable testing conditions, such as by modifying the type of memory test and/or prior success in cognitive tasks. That work might also compare the naturalistic stereotype presentation to traditional age-based stereotype threat inductions, separately and in combination. Such detailed follow-up examinations would help “unpack” findings from this novel approach employing more naturalistic exposure to stereotypes.

However, the absence of a threat reaction to the naturalistic stereotype manipulation or potentially minimized “threat in the air” from the favorable testing conditions in this study cannot explain the superior memory performance of those in the stereotype group compared to the control group. A few trends in our data hint at factors that might partially explain or moderate the observed benefits related to the stereotype activation. First, the effect might represent stereotype lift if the participants do not identify “old” as self-relevant (Walton and Cohen, 2003). This effect may be particularly likely for participants whom we classified as “late middle-aged” but may apply to “older adults” who do not identify as old. Although the age group × condition interaction was not significant, follow-up analysis suggested superior memory in the stereotype group compared to the control group for late middle-aged participants, but no difference in performance between the two conditions for older participants. In some research, the young-old have been most vulnerable to age stereotypes with middle-aged and older-old adults showing resilience (Hess et al., 2004; Eich et al., 2014). In this case, we speculate that late middle-aged participants may have been motivated to perform better following presentation of negative age and memory stimuli. That is, middle-aged participants’ performance may have been boosted from the positive downward social comparison of themselves to those who are old. In support of this stereotype lift interpretation, and consistent with other research (Rubin and Berntsen, 2006), participants in this study reported subjective ages that were, on average, 21% younger than their actual ages (equivalent to a 65-year-old feeling 51 years old). The rejection of personal relevance of the stereotype stimuli may reflect age-group dissociation reported by others in terms of feeling younger and having lower identification with “old” following stereotype manipulation (Weiss and Freund, 2012; Weiss and Lang, 2012). One interesting avenue for future research would be systematic comparison of negative versus neutral age stereotype activation on memory performance for adults of varied chronological ages who self-identify as “old” or “middle-aged” prior to the experiment. Unlike many other personal features that are stereotyped, this would be particularly valuable for understanding age stereotype activation given that the age identification process evolves over time.

The observed age-group dissociation effects might suggest the naturalistic stereotype manipulation functioned as a stereotype priming effect, promoting stereotype assimilation or embodiment. However, we did not observe differences between the stereotype group and control group in reported subjective age and memory evaluation following the stereotype manipulation and memory testing, which we proposed would suggest stereotype embodiment. Instead, we noted that participants who reported awareness of the stereotype stimuli trended toward a more positive evaluation of their memory than those who were unaware of the stimuli, a possible sign of stereotype lift or age-group dissociation. That is, when aware of the stereotype presentations, participants may have rejected their self-relevance, reporting more positive memory evaluations (inconsistent with old age stereotypes) rather than reporting poorer memory evaluations, which would suggest stereotype assimilation. This evidence complements findings of other research emphasizing the difference in impact of subtle or implicit versus blatant or explicit stereotype manipulations (Horton et al., 2008; Lamont et al., 2015). For example, the impact of stereotype stimuli presented blatantly (e.g., highlighted in yellow) in a lexical decision task on memory performance was lesser than the impact of the same stimuli presented less obviously (e.g., not highlighted), and age differences in memory performance (between younger and older adults) were exaggerated following subtle, rather than blatant, presentation of negative age stereotypes (Hess et al., 2004). Furthermore, our finding aligns with Weiss and Kornadt (2018) observations that blatant stereotype manipulations may promote age-group dissociation whereas subtle stereotype manipulations promote stereotype internalization.

In sum, our findings suggest that the presentation of negative age and memory stereotypes could bolster the memory performance of late middle-aged adults, and perhaps older adults, specifically when (a) memory testing follows a “success” experience for a different cognitive task, (b) conditions of the testing situation are generally favorable, and (c) participants’ overall perception of stereotype threat is not high. The findings of this research are notable because they evidence better behavioral performance following a negative stereotype condition, rather than a positive stereotype condition, as in Swift et al. (2013) or Meisner (2012). Collectively, our results did not support the idea that the naturalistic stereotype presentation could induce age-based stereotype threat. However, it is possible for older adults–who had similar performance in the stereotype condition and control condition–that the supportive conditions of our testing situation nullified potential detrimental impact of the stereotype presentation. This notion is partially supported by the marginally significant evidence for moderation of the stereotype effect by perceived stereotype threat. Given our relatively selective sample (e.g., healthy and well-educated) a direct replication of our findings by others is warranted. We also recommend that future research test dispositional and state perceptions of stereotype threat, as in Kang and Chasteen (2009), to separately evaluate threat felt in response to manipulations and individual differences in overall sensitivity to stereotype threat.

Furthermore, we suggest additional research examine the role of memory beliefs as potential moderators of reactions to stereotype presentations. In this study, participants who reported awareness of the stereotype stimuli might have been motivated to disprove the stereotypes (as suggested by hints of more positive memory evaluation compared to the unaware group), or exert their “free will” as in stereotype reactance theory (Miron and Brehm, 2006). Perhaps memory beliefs could help explain responses consistent with stereotype assimilation versus those aligned with group dissociation. For example, memory beliefs may moderate responses to blatant age stereotypes: If one has high confidence in their memory or believes their memory performance is within their control, they may be more emboldened to “prove wrong” (or prove personally irrelevant) stereotypes they notice about senility in aging. Yet, these beliefs might not be able to protect individuals from subtly presented stereotypes that they do not consciously notice. Certainly, the results here do not suggest that negative age and memory stereotypes should be promoted to benefit late middle-aged people. Such benefits might be short-lived or cognitively taxing, and adoption of negative attitudes about aging earlier in life may be related to a host of negative aging outcomes later in life, when the stereotypes eventually become self-relevant, and result in more problems later (Levy, 2009; Weiss and Kornadt, 2018).

Instead, an interesting series of follow-up studies might emphasize trying to train awareness and responses to the stereotype primes, while also promoting more positive attitudes toward aging and better memory beliefs. The latter component may be especially important given that stereotype priming can promote stereotype-consistent behavior even in outgroups, especially if they personally associate the stereotyped group and the characteristics (Wheeler and Petty, 2001). To better tease out how age is related to type of stereotyped stimuli and testing environments, more stereotype activation work should aim to test for the real world, practical impact of age and memory stereotypes on performance “in the wild.” Such examinations might explicitly compare such results to those observed in environments with varying degrees of possible threat (i.e., number of opportunities for success before memory testing, emphasis on knowledge or expertise, degree of familiarity of stimuli in which stereotypes are embedded, ecological validity of specific memory tests). Systematic follow-up studies, using a wide range of age groups, would further help researchers to identify the specific mechanisms controlling stereotype lift effects.

## Data Availability Statement

The raw data supporting the conclusions of this article will be made available by the authors, without undue reservation.

## Ethics Statement

The studies involving human participants were reviewed and approved by University of Florida Institutional Review Board 02 (approval code #2015-U-0680). The patients/participants provided their written informed consent to participate in this study.

## Author Contributions

CS-H took the lead in analysis and interpretation of data and drafting of the manuscript in consultation with RW. Both authors conceived and planned the study, supervised the data collection, provided critical feedback, and helped to shape the research, analysis and manuscript.

## Conflict of Interest

The authors declare that the research was conducted in the absence of any commercial or financial relationships that could be construed as a potential conflict of interest.

## Supplementary Material

Supplementary Data Sheet 1 Word search and word jumble puzzles used for the stereotype activation.

Supplementary Data Sheet 2 Reference sheet provided to aid completion of the word search and word jumble puzzles.
