# Supplementary material for: The Impact of Naturalistic Age Stereotype Activation
Source: Front Psychol. 2021 Jul 9;12:685448. doi: 10.3389/fpsyg.2021.685448 (PMC8302256; doi:10.3389/fpsyg.2021.685448)
Supplement: Supplementary Data Sheet 1 — Word search and word jumble puzzles used for the stereotype activation. [file Data_Sheet_1.PDF]

Word Search Puzzle – Control Condition – Example (WS-C-X)

Instructions: Find the words from the list hidden in the puzzle. Feel free to refer back to your instruction sheet as you work on this activity.

W	J	I	S	R	B
D	A	O	N	O	M
P	S	I	U	A	N
E	A	P	T	D	B
T	C	E	B	E	O
N	I	B	A	C	R

CABIN	ROAD	WAITER

Word Search Puzzle – Control Condition – Version A (WS-C-A)

Instructions: Find the words from the list hidden in the puzzle. Feel free to refer back to your instruction sheet as you work on this activity.

W	T	K	U	X	O	C	R	I	K
A	Z	N	D	B	H	E	O	N	O
R	E	C	A	S	V	N	X	I	X
R	G	S	T	I	A	I	U	E	N
I	D	U	R	F	G	Z	G	J	Z
O	I	A	N	I	M	A	L	S	I
R	R	D	K	H	L	G	G	L	V
I	B	Z	I	W	L	A	C	D	E
W	I	N	D	O	W	M	C	X	J
A	C	J	Y	R	E	T	T	E	L

ANIMALS	GIANT	RIVER
BRIDGE	LETTER	WARRIOR
COIN	MAGAZINE	WINDOW

Word Search Puzzle – Control Condition – Version B (WS-C-B)

Instructions: Find the words from the list hidden in the puzzle. Feel free to refer back to your instruction sheet as you work on this activity.

C	F	A	T	R	A	E	H	E	T
H	L	M	A	C	H	I	N	E	N
A	H	I	E	W	E	O	R	B	E
P	T	L	N	S	D	X	A	A	R
T	F	R	C	N	U	S	N	U	A
E	C	O	F	O	E	O	S	T	P
R	T	A	R	B	H	S	H	H	C
G	E	M	A	E	E	R	G	O	G
S	C	L	A	O	S	H	C	R	E
B	L	N	O	O	M	T	P	T	N

AUTHOR	FOREST	MACHINE
BASEBALL	HEART	MOON
CHAPTER	HOUSE	PARENT

Word Search Puzzle – Stereotype Condition – Example (WS-ST-X)

Instructions: Find the words from the list hidden in the puzzle. Feel free to refer back to your instruction sheet as you work on this activity.

R	J	I	S	A	B
D	E	O	N	G	M
P	S	C	U	E	N
E	A	P	A	D	B
T	C	E	B	L	O
H	C	T	A	W	L

AGED	RECALL	WATCH

Word Search Puzzle – Stereotype Condition – Version A (WS-ST-A)

Instructions: Find the words from the list hidden in the puzzle. Feel free to refer back to your instruction sheet as you work on this activity.

Q	W	E	A	K	T	L	T	V	F
W	D	I	A	E	P	E	R	R	C
O	X	E	I	L	E	X	A	J	O
L	Z	X	M	E	N	G	G	U	N
L	E	Y	L	E	I	O	X	M	F
I	L	B	F	L	N	F	L	H	U
P	A	N	E	O	C	T	Q	Z	S
T	O	I	P	A	I	O	I	X	E
Y	A	A	V	E	N	U	E	A	D
R	K	L	A	R	E	T	T	I	B

AVENUE	DEMENTIA	PILLOW
BITTER	FRAGILE	TABLE
CONFUSED	INEPT	WEAK

Word Search Puzzle – Stereotype Condition – Version B (WS-ST-B)

Instructions: Find the words from the list hidden in the puzzle. Feel free to refer back to your instruction sheet as you work on this activity.

B	E	M	R	O	F	I	N	U	Q
M	C	H	E	L	P	L	E	S	S
L	U	O	T	M	F	V	L	K	I
K	I	N	F	R	T	L	I	Z	M
L	A	A	A	F	M	O	N	X	P
M	S	I	C	R	E	N	E	L	A
C	L	T	W	H	W	E	S	O	I
D	K	R	O	N	Z	L	R	F	R
G	A	H	F	N	U	Y	T	P	E
T	S	O	L	B	E	W	H	I	D

COFFEE	IMPAIRED	STONE
FRAIL	LONELY	SENILE
HELPLESS	LOST	UNIFORM

Word Jumble Puzzle – Example (J-X)

Instructions: First, solve the jumbled words. Unscramble them to form a word from the list. Then, use the circled letters from your answers to fill in the surprise answer at the end. The surprise answer is suggested by the cartoon. Feel free to refer back to your instruction sheet as you work on this activity.

ACEK

NIKP

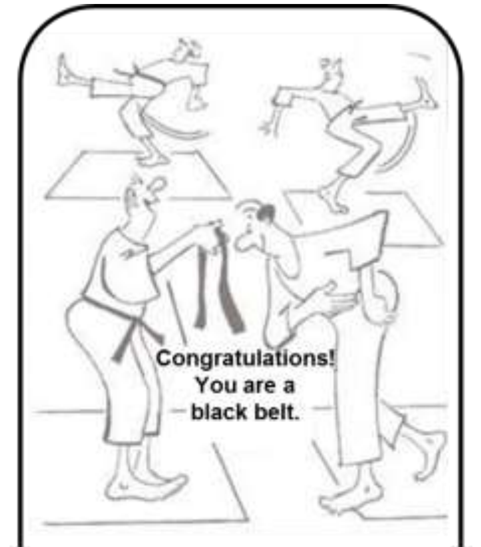

WHAT HE GOT WHEN HE TOOK KARATE LESSONS

A: _ _ _ _ _ OUT OF IT

CAKE	EXIT	PINK	PUFF

Word Jumble Puzzle – Control Condition – Version A (J-C-A)

Instructions: First, solve the jumbled words. Unscramble them to form a word from the list. Then, use the circled letters from your answers to fill in the surprise answer at the end. The surprise answer is suggested by the cartoon. Feel free to refer back to your instruction sheet as you work on this activity.

EIRKB

RAORBH

APDCLE

UUYLNR

IHGUBNSR

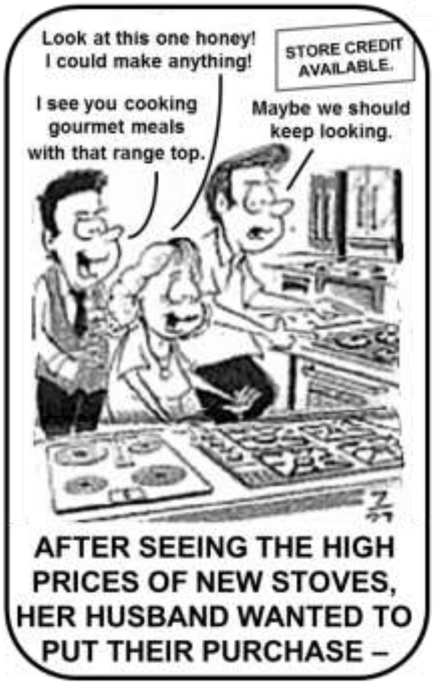

A: ON THE

AGAIN	BIKER	BRUSHING	BUSTED	FLIGHT
HARBOR	HEATED	IMPORTED	PLACED	UNRULY

Word Jumble Puzzle – Control Condition – Version B (J-C-B)

Instructions: First, solve the jumbled words. Unscramble them to form a word from the list. Then, use the circled letters from your answers to fill in the surprise answer at the end. The surprise answer is suggested by the cartoon. Feel free to refer back to your instruction sheet as you work on this activity.

ANAGI

TEBSDU

HGLIFT

ETEADH

RODPTIEM

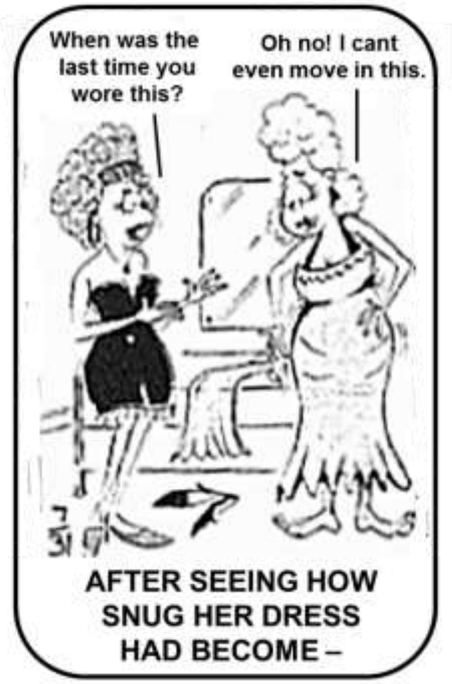

A: ___ WAS _______

AGAIN	BIKER	BRUSHING	BUSTED	FLIGHT
HARBOR	HEATED	IMPORTED	PLACED	UNRULY

Word Jumble Puzzle – Stereotype Condition – Version A (J-ST-A)

Instructions: First, solve the jumbled words. Unscramble them to form a word from the list. Then, use the circled letters from your answers to fill in the surprise answer at the end. The surprise answer is suggested by the cartoon. Feel free to refer back to your instruction sheet as you work on this activity.

RAEPP

GNADIF

RNERNU

ERITER

SMPALIEC

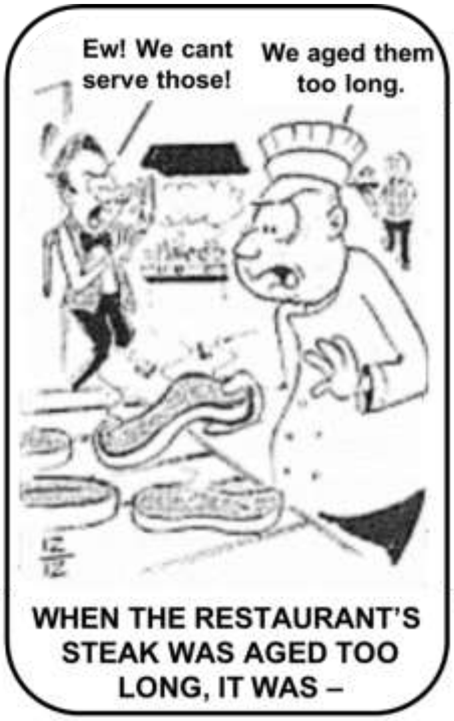

A: ◯◯◯◯ ITS ◯◯◯◯◯

AFTER	DECLINED	FADING	FORGOT	HAMMER
MISPLACE	PAPER	RETIRE	RUNNER	SENIOR

Word Jumble Puzzle – Stereotype Condition – Version B (J-ST-B)

Instructions: First, solve the jumbled words. Unscramble them to form a word from the list. Then, use the circled letters from your answers to fill in the surprise answer at the end. The surprise answer is suggested by the cartoon. Feel free to refer back to your instruction sheet as you work on this activity.

TAREF

TROOGF

MMEARH

ISRONE

NEDIELCD

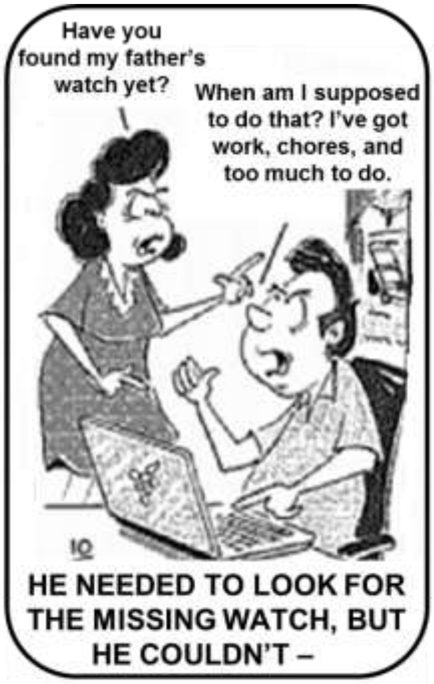

A: ○○○○ THE ○○○○

AFTER	DECLINED	FADING	FORGOT	HAMMER
MISPLACE	PAPER	RETIRE	RUNNER	SENIOR
