# Supplementary material for: The Impact of Naturalistic Age Stereotype Activation
Source: Front Psychol. 2021 Jul 9;12:685448. doi: 10.3389/fpsyg.2021.685448 (PMC8302256; doi:10.3389/fpsyg.2021.685448)
Supplement: Supplementary Data Sheet 2 — Reference sheet provided to aid completion of the word search and word jumble puzzles. [file Data_Sheet_2.PDF]

WORD SEARCH INSTRUCTIONS

The goal of Word Search is to find the words hidden within the puzzle. The list below the puzzle will show you all the words to find. Search **up, down, forward, backward,** and on the **diagonals** to find the hidden words. When you find a word, circle it in the puzzle. Then, make sure to cross it off the list!

Try the tips and tricks below to make the puzzle easier.

TIPS AND TRICKS

- Check the edges of the word search box. Many times there will be words along the edges.
- Longer words tend to be on the diagonal.
- Look for the first letter in a word by searching back-and-forth along each row, from top to bottom. Or, try going down each column.
- Use another piece of paper or your pencil and hold it next to each line, or each diagonal, as you check it – this will help you keep your eyes on that one line or diagonal as you look for words.
- Try to search for these letters or features that are easier to find:
  - Less-common letters, such as ***J, B, K, Q, X, Y,*** or ***Z***
  - Double letters, such as ***OO, EE,*** or ***TT***
  - Circular letters, especially ***O, D,*** and ***Q***.
- When you find a letter, do a *circle search* around it to look for the next letter(s) in the word.
- Look at your word list to see if one or two letters occur more frequently (e.g., several **R**'s in the word list). If there is a specific letter or word pattern (***OR, DE***) that occurs frequently in the list, search for that one letter or pattern first because it may help you to find several words.

JUMBLES INSTRUCTIONS

The first goal of Jumbles is to rearrange or unscramble letters to form words. Each scramble will form an ordinary word. For example, the letters in **EKCN** could be rearranged to form the word **NECK**. Each box needs only one letter.

The second goal of Jumbles is to solve the surprise answer that is suggested by the cartoon. After you unscramble several words, you arrange the circled letters to form the surprise answer at the bottom. Each circled letter will be used only once in the surprise answer.

Because some people do not solve Jumbles regularly, we have also included a word list to assist you. All of the unscrambled word answers (but not the surprise answer) can be found in the list, along with some additional words.

Try the tips and tricks below to make the Jumble puzzle easier.

TIPS AND TRICKS

- The surprise answer is often a joke or pun.
- Look for any letters that appear frequently together. These include consonants such as ***CH, ST, PL*** or vowel consonant combinations like ***QU*** or ***ED***.
- Some Letter Patterns tend to be found at the start or end of a word (see box on right). Look for these patterns as you try to identify the scrambled words.
- Use scratch paper to rewrite the scrambled letters:
    - Write out each consonant with a different vowel
    - Write the letters down like the numbers on the face of a clock
    - Try writing the letters in different orders (for example, EAQKSU might be easier if seen as SEA QUK)
    - Find letters that can be used to form a Letter Pattern shown in the box (the Start and End letters). Then write the other letters before or after the letter pattern you found.
- You may be able to fill in the surprise answer before unscrambling all the words. If you do that, then you can work backwards – use the letters in the surprise answer to help identify the circled letters in the jumbled words.

Letter Patterns	
**Start**	**End**
DE -	- ED
IM -	- ER
MIS -	- FUL
OUT -	- ING
RE -	- LY
UN -	- S
UP -	- Y
